# New Trauma and Injury Severity Score (TRISS) adjustments for survival prediction

**DOI:** 10.1186/s13017-018-0171-8

**Published:** 2018-03-06

**Authors:** Cristiane de Alencar Domingues, Raul Coimbra, Renato Sérgio Poggetti, Lilia de Souza Nogueira, Regina Marcia Cardoso de Sousa

**Affiliations:** 1 All Trauma, São Paulo, SP Brazil; 2 University of California San Diego Medical Center, San Diego, CA USA; 3 Medical School, University of Sao Paulo, São Paulo, SP Brazil; 4 School of Nursing, University of Sao Paulo, São Paulo, SP Brazil

**Keywords:** Wounds and injuries, Injury Severity Score, Traumatology, Outcome assessment

## Abstract

**Background:**

The objective of this study is to propose three new adjustments to the Trauma and Injury Severity Score (TRISS) equation and compare their performances with the original TRISS as well as this index with coefficients adjusted for the study population.

**Methods:**

This multicenter, retrospective study evaluated trauma victims admitted to two hospitals in São Paulo-Brazil and San Diego-EUA between January 1st, 2006, and December 31st, 2010. The proposed models included a New Trauma and Injury Severity Score (NTRISS)-like model that included Best Motor Response (BMR), systolic blood pressure (SBP), New Injury Severity Score (NISS), and age variables; a TRISS peripheral oxygen saturation (SpO_2_) model that included Glasgow Coma Scale (GCS), SBP, SpO_2_, Injury Severity Score, and age variables; and a NTRISS-like SpO_2_ model that included BMR, SBP, SpO_2_, NISS, and age variables. All equations were adjusted for blunt and penetrating trauma coefficients. The model coefficients were established by logistic regression analysis. Receiver operating characteristic (ROC) curve analysis was used to evaluate the performance of the models.

**Results:**

The original TRISS (area under the curve (AUC) = 0.90), TRISS with adjusted coefficients (AUC = 0.89), and the new proposals (NTRISS-like, TRISS SpO_2_, and NTRISS-like SpO_2_) showed no difference in performance (AUC = 0.89, 0.89, and 0.90, respectively).

**Conclusions:**

The new models demonstrated good accuracy and similar performance to the original TRISS and TRISS adjusted for coefficients in the study population; therefore, the new proposals may be useful for the assessments of quality of care in trauma patients using variables that are routinely measured and recorded.

## Background

The quality of trauma care is assessed by the Performance Improvement and Patient Safety (PIPS) Program, based on trauma records and severity indexes [1]. Several severity scoring systems are available; some are universally accepted and reviewed periodically in order to improve their accuracy. These include the Trauma and Injury Severity Score (TRISS), a tool well suited for the evaluation of the quality of care and to propose improvements in trauma care [2]. The predictive value of the TRISS can be maximized by adjusting for coefficients in the population in which it is being applied [3–8].

The TRISS comprises the Revised Trauma Score (RTS) and Injury Severity Score (ISS) indexes as well as the trauma type (blunt or penetrating) and the patient age. Although the TRISS is widely used, it presents limitations which involve, mainly, the RTS and the ISS [9, 10].

Currently, the RTS is difficult to calculate due to increases in the number of rapid-sequence endotracheal intubations performed in prehospital setting, an intervention that makes it impossible to determine the Glasgow Coma Scale (GCS) score and respiratory rate (RR) upon hospital admission, which are necessary for the calculation of the RTS. In addition, the RR is a physiological parameter that requires time to measure during the emergency care of trauma patients. Its normal range is very broad and abnormal values may not be directly related to respiratory function deficits [11]. Peripheral oxygen saturation (SpO_2_) has gained a place as a respiratory parameter in emergency situations as it allows the evaluation of the tissue perfusion quality in trauma patients and is quick and easy to measure. Regarding the GCS, the literature proposes to replace the total score of the scale with the value of the Best Motor Response (BMR) item [12].

The ISS component has been criticized for not considering more than one lesion in each body region in its calculation, which may underestimate the severity [13–16]. The updated ISS, the New Injury and Severity Score (NISS), considered the three most serious injuries in calculating the severity of the trauma, regardless of the body region affected, thus seeking to increase the sensitivity of the index, as trauma patients can present multiple severe injuries in the same body region [13–16].

As a result of these criticisms, several proposals to modify the TRISS have been published; however, studies that replace the ISS by the NISS or which include the SpO_2_ in its components are scarce [12]. The present study presents three new proposals—New Trauma and Injury Severity Score (NTRISS)-like, TRISS SpO_2_, and NTRISS-like SpO_2_. The first new variation (NTRISS-like) combines the physiological BMR parameters of the GCS, systolic blood pressure (SBP), and the anatomical variable of the NISS. The second and third variations include SpO_2_. In the TRISS SpO_2_, the RTS is replaced by GCS and SBP values and SpO_2_ score; in the NTRISS-like SpO_2_, the value assigned to SpO_2_ was added to the NTRISS-like index.

Thus, the objective of this study was to compare the accuracy of three proposed variations to the original TRISS index with coefficients adjusted for the study population in predicting survival and to verify the viability of these new proposals as a replacement of the TRISS.

## Methods

This multicenter, retrospective cohort study was performed in two reference hospitals for trauma care, one in the city of São Paulo, Brazil (SPBRA), and another in San Diego County, USA (SDEUA).

The study included 10,588 patients, 2416 hospitalized at the SPBRA emergency room and 8172 at the SDEUA trauma center for traumatic and/or penetrating traumatic events, aged 14 years or more between January 1, 2006, and December 31, 2010. The traumatic events were those listed in Chapter XX of the International Statistical Classification of Diseases and Health Related Problems (ICD-10), excluding cases of hanging, suffocation, drowning or near drowning, poisoning, burning, and electrocution. Patients admitted after the first 24 h of the traumatic event or transferred from other hospitals were excluded from the study since the calculation of survival probability by the models requires information from the patient’s initial clinical condition.

A total of 300 patients from each of the institutions were randomly selected from the database (10,588 patients), which contained all the information required for the calculation of the indexes of survival probability; this Test Database was used to assess the accuracy of the models. Data from the other patients were grouped in the Derived Database and used to identify the coefficients of the proposed models (NTRISS-like, TRISS SpO_2_, and NTRISS-like SpO_2_), as well as adjust the weightings of the TRISS to the study population for both blunt and penetrating trauma.

To ensure that the two hospitals had the same importance in the derivation of the coefficients, a weight of 3.72 was given to each of the SPBRA patients due to the disproportionate distributions of patients from this institution and SDEUA in the Derived Database (2116 versus 7872).

All models of survival probability compared in this study used physiological parameters obtained upon patient hospital admission and were calculated by the equation Ps = 1/(1 + *e*^−*b*^), in which:

Ps, probability of survival

*e*, 2.718282 (base of the Neperian logarithm).

The values of *b* differed in all three models, as follows:

TRISS

$$ b={b}_0+{b}_1\left(\mathrm{RTS}\right)+{b}_2\left(\mathrm{ISS}\right)+{b}_3\left(\mathrm{age}\right) $$

RTS, total value of the index (0 to 12)

Age, 0 if age < 55 years and 1 if age ≥ 55 years.

NTRISS-like

$$ b={b}_0+{b}_1\left(\mathrm{BMR}\right)+{b}_2\left(\mathrm{SBP}\right)+{b}_3\left(\mathrm{NISS}\right)+{b}_4\ \left(\mathrm{age}\right) $$

BMR, value attributed to this item in the GCS (1 to 6)

SBP, value assigned to this parameter in the RTS (0 to 4)

Age, 0 if age < 55 years and 1 if age ≥ 55 years.

TRISS SpO_2_

$$ b={b}_0+{b}_1\left(\mathrm{GCS}\right)+{b}_2\left(\mathrm{SBP}\right)+{b}_3\left({\mathrm{SpO}}_2\right)+{b}_4\left(\mathrm{ISS}\right)+{b}_5\ \left(\mathrm{age}\right) $$

GCS and SBP, values assigned to these parameters in the RTS (0 to 4)

SpO_2_, according to the following values, 0 or not measurable = 0; 1 to 80 = 1; 81 to 90 = 2; 91 to 95 = 3; 96 to 100 = 4

Age, 0 if age < 55 years and 1 if age ≥ 55 years.

NTRISS-like SpO_2_

$$ b={b}_0+{b}_1\left(\mathrm{BMR}\right)+{b}_2\left(\mathrm{SBP}\right)+{b}_3\left({\mathrm{SpO}}_2\right)+{b}_4\left(\mathrm{NISS}\right)+{b}_5\ \left(\mathrm{age}\right) $$

BMR, used the value attributed to this item on the GCS (1 to 6)

SBP, value assigned to this parameter in the RTS (0 to 4)

SpO_2_, according to the following values, 0 or not measurable = 0; 1 to 80 = 1; 81 to 90 = 2; 91 to 95 = 3; 96 to 100 = 4.

Age, 0 if age < 55 years and 1 if age ≥ 55 years.

The coefficients of the proposed models and TRISS adjusted to the study population were derived by logistic regression. The diagnostic test receiver operating characteristic (ROC) was used to evaluate the predictive capacity of the new models and the original and adjusted TRISS.

## Results

In the sample of 10,588 patients attended between January 1, 2006, and December 31, 2010, was a predominance of males (73.5%). Transportation accidents (44.1%), falls (30.3%), and assaults (18.0%) were the most common external causes. Blunt trauma mechanism was the most common (90.4%). A total of 2736 victims (25.8%) underwent surgical procedures and 4132 patients (39.0%) were admitted to the Intensive Care Unit. The patients remained hospitalized for an average of 5.4 ± 13.3 days and mortality was 5.9% (Table 1).

**Table 1:** Descriptive statistics related to trauma and patient gender, age, and clinical variables. São Paulo–San Diego, 2006–2010

Variables	*N* (%)
Age (years), mean (SD)	41.9 (± 19.9)
Gender	
Male	7798 (73.5)
Female	2790 (26.5)
Mechanism of trauma	
Blunt	9570 (90.4)
Penetrant	1016 (9.6)
No information	2 (0.0)
External causes of morbidity and mortality	
Transportation accidents	4663 (44.1)
Falls	3203 (30.3)
Assault	1908 (18.0)
Intentional self-harm	216 (2.0)
Events with undetermined intent	205 (1.9)
Other	364 (3.4)
No information	29 (0.3)
Surgical procedure	
Yes	2736 (25.8)
No	7852 (74.2)
ICU admission	
Yes	4132 (39.0)
No	6456 (61.0)
Hospital discharge condition	
Survived	9962 (94.1)
Died	626 (5.9)
Hospital length of stay (days), mean (SD)	5.4 (± 13.3)
ISS, mean (SD)	9.7 (± 9.6)
NISS, mean (SD)	12.8 (± 13.0)

*ICU* Intensive Care Unit, *ISS* Injury Severity Score, *NISS* New Injury Severity Score

Considering the physiological variables, 82.8% presented a GCS score between 13 and 15; the mean SBP was 133.7 ± 31.7 mmHg, and the mean RR was 18.1 ± 5.2 breathes per minute. Peripheral oxygen saturation (SpO_2_) presented an average value of 97.3 ± 8.9%. Only 459 (4.3%) victims did not present GCS or BMR values at hospital admission, and SBP information was missing from 0.9% of the study population. SpO_2_ and RR data were missing from 29.6 and 8.3% of the victims, respectively. The mean RTS was 7.4 ± 1.4.

The patients presented a mean of 2.1 ± 1.0 injured body regions, the most commonly affected external surfaces being the head (1.6 ± 1.4 injuries) and neck (1.0 ± 1.4 injuries). The mean ISS and NISS values were 9.7 ± 9.6 and 12.8 ± 13.0, respectively. It was not possible to calculate the ISS and NISS in 48 victims (0.5%).

The coefficients for blunt and penetrating trauma of the new models (NTRISS-like, TRISS SpO_2_, and NTRISS-like SpO_2_) and adjusted TRISS are shown in Table 2.

**Table 2:** Coefficients of adjusted TRISS, NTRISS-like, TRISS SpO_2_, and NTRISS-like SpO_2_ derived from the Derived Database for blunt and penetrating trauma. São Paulo–San Diego, 2006–2010

Adjusted TRISS 1/(1 + *e*^−*b*^), where *b* = *b*_0_ + *b*_1_(RTS) + *b*_2_(ISS) + *b*_3_(age)*		
Coefficients	Blunt	Penetrating
*b*_*0*_	− 1.64790049	− 1.29803310
*b*_1_	0.90535734	0.89538700
*b*_2_	− 0.07845091	− 0.09521947
*b*_3_	− 1.38013670	− 1.27540759
NTRISS-like 1/(1 + *e*^−*b*^), where *b* = *b*_0_ + *b*_1_(BMR) + *b*_2_(SBP) + *b*_3_(NISS) + *b*_4_ (age)*		
Coefficients	Blunt	Penetrating
*b*_0_	− 1.67602650	− 1.58632944
*b*_1_	0.61944706	0.58883203
*b*_2_	0.89539814	0.96952677
*b*_3_	− 0.07289039	− 0.06659814
*b*_4_	− 1.33088941	− 1.00582810
TRISS SpO_2_ 1/(1 + *e*^−*b*^), where *b* = *b*_0_ + *b*_1_(GCS) + *b*_2_(SBP) + *b*_3_(SpO_2_) + *b*_4_(ISS) + *b*_5_(age)*		
Coefficients	Blunt	Penetrating
*b*_0_	− 2.97523446	− 3.5166820
*b*_1_	0.75773826	0.8515884
*b*_2_	0.58321377	0.3453793
*b*_3_	0.38492625	1.3098071
*b*_4_	− 0.08441861	− 0.1955984
*b*_5_	− 1.59455370	− 4.0353761
NTRISS-like SpO_2_ 1/(1 + *e*^−*b*^), where *b* = *b*_0_ + *b*_1_(BMR) + *b*_2_(SBP) + *b*_3_(SpO_2_) + *b*_4_(NISS) + *b*_5_ (age)*		
Coefficients	Blunt	Penetrating
*b*_0_	− 2.73634921	− 1.5156694
*b*_1_	0.59396868	0.1832071
*b*_2_	0.66226833	1.0209288
*b*_3_	0.56405908	1.1288631
*b*_4_	− 0.06841853	− 0.1138697
*b*_5_	− 1.43274160	− 1.7286860

*BMR* Best Motor Response, *SBP* systolic blood pressure, *NISS* New Injury Severity Score, *GSC* Glasgow Coma Scale, *ISS* Injury Severity Score, *SpO*_*2*_ peripheral oxygen saturation

*Age, 0 if < 55 years; 1 if ≥ 55 years

**Fig. 1:**
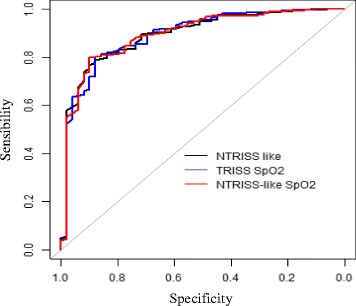
ROC curves of NTRISS-like, TRISS SpO2, and NTRISS-like SpO2 in predicting survival. São Paulo–San Diego, 2006–2010

Figure 1 shows the ROC curves of the new models. The curves of the three indexes overlap, indicating a similarity between them in the prediction of survival.

The accuracy of all models analyzed in this study was high (area under the curve above 0.89). In addition, the confidence intervals (CIs) of all areas under the ROC curve were similar (95% CI 0.85–0.94) (Table 3).

**Table 3:** Predictive ability of original TRISS, adjusted TRISS, and new models. São Paulo–San Diego, 2006–2010

	Sens. (%)	Spec. (%)	Cutoff	CI 95%	AUC
TRISS	80.2	83.7	0.97	0.85–0.94	0.90
Adjusted TRISS	82.6	81.6	0.95	0.85–0.94	0.89
NTRISS-like	78.9	87.8	0.96	0.85–0.94	0.89
TRISS SpO_2_	80.4	87.8	0.96	0.85–0.94	0.89
NTRISS-like SpO_2_	79.9	89.8	0.97	0.85–0.94	0.90

*Sens.* sensibility, *Spec.* specificity, *CI* confidence intervals, *AUC* area under the curve

## Discussion

Given the criticism of the component variables of the TRISS, the new proposals included GCS or BMR, SBP, and SpO_2_ as variables in the models and excluded RTS and RR from the regression equation, as these parameters exclude the presumably more serious patients from the analysis of survival probability (intubated) [10, 17, 18]. A literature review of comparative studies of the original TRISS with modified models showed an improvement in TRISS performance when the RTS was removed from the model and replaced by the GCS, BMR, SBP, and RR parameters directly in the equation [12].

In view of the indications in the literature that the regression coefficients should be adjusted for the individual site, the results of the present study verified that the adjustment of the weightings to the study population improved the predictive capacity of the TRISS and that this capacity was maintained, as in other studies [6–19].

A literature review of studies that made adjustments to the original TRISS equation and compared the discriminatory capacity of the modified equation with the original in the prediction of survival showed that adjustments of the coefficients in the index equation were frequent. However, the results showed no trend of improvement in the performance of the models with this type of modification, with a performance improvement reported in only 30% of the analyzed studies [12].

The changes of physiological variables proposed in the new models did not increase the predictive capacity in comparison to the original and adjusted TRISS. The NTRISS-like model, a variation of the TRISS-like model that replaces the ISS was with the NISS, had a similar performance to TRISS. The TRISS-like model was introduced in 1992 [10] and includes only the BMR physiological parameters of the GCS and SBP in order to evaluate potentially more serious patients (intubated) in the calculation of the index; however, this model was criticized in the literature [11, 20] for removing the respiratory parameter from the evaluation of the survival probability. The TRISS-like model also showed a similar performance to the TRISS [10, 17].

The introduction of the SpO_2_ in the NTRISS-like index (NTRISS-like SpO_2_) did not improve the performance of the model, indicating that SpO_2_ as an isolated adjustment did not increase the predictive ability of this model. A study that evaluated the role of the RR and SpO_2_ in the mortality of trauma patients reported that these two parameters were not good predictors for this outcome when added separately to the TRISS equation (RTS with neutralized RR + ISS + age + RR or RTS with neutralized RR + ISS + age + SpO_2_) [11].

Proposals for replacement of the RTS by physiological parameters such as GCS, BMR, SBP, and RR in the equation resulted, in general, in an equivalent or better performance compared to the original TRISS [12]; in the present study, the substitution of different physiological variables in the models NTRISS-like, TRISS SpO_2_, and NTRISS-like SpO_2_ also resulted in an equivalent predictive value to that of the original TRISS.

Regarding the anatomical variable, replacement of the ISS by the NISS in the proposed formulas also resulted in a similar performance to that of the original TRISS and the new model that retained the ISS in the equation (TRISS SpO_2_). Of four published studies [16, 21–23], which replaced the ISS by the NISS in the TRISS equation, only one [21] showed an improved performance of the index without this adjustment.

The ISS considers the three most serious lesions in different body regions of the victim [24], while the NISS [13] includes the three most serious lesions, regardless of the affected region. Due to the similarity of the performance of the proposed models using the ISS or NISS in the equations and the advantage of the ease of calculation of the NISS, this index is proposed in the survival models.

In this study, the original TRISS with adjusted coefficients and the new proposals had similar performances and accuracies between 89.0 and 90.0%. In the literature, the accuracies of the studies that showed no difference between the predictive power of the TRISS and the new proposals ranged from 85.3 to 96.4% [6, 11, 22, 25]. The studies in which the adjustments of the TRISS resulted in improved predictive ability presented higher accuracies, ranging between 90.1 and 98.1% [4, 6, 16, 25–28].

Although they do not improve the predictive accuracy of the TRISS, the models proposed in this study were equivalent and, given the clinical significance and ease of obtaining information from its components, seem to be good options to estimate the survival probability of trauma victims.

One limitation of this study is that the frequent loss (29.6%) of SpO_2_ value may have negatively influenced the predictive capacity of the models that used this parameter (TRISS SpO_2_ and NTRISS-like SpO_2_). The inclusion of SpO_2_ in these models had as a premise the improvement in the performance of the TRISS, considering a probable higher availability of this information compared to the RR and the potential of this parameter to contribute to the estimation of the severity of the physiological conditions of the patient.

Nevertheless, the frequent lack of SpO_2_ data may have underestimated its importance in the survival prediction models. While SpO_2_ is a procedure performed in emergency services, the results are not always registered; once the inclusion of this variable in the calculation of the indexes is established, the loss of these will likely decrease.

Although they were derived using the variable with the highest degree of data loss, the TRISS SpO_2_ and NTRISS-like SpO_2_ had equivalent predictive values to that of the other indexes. Thus, the two models that include SpO_2_ may also be recommended due to the clinical ease in obtaining SpO_2_ and its physiological significance, since they reflect both oxygenation and circulation, while the RR reflects only ventilation [11]. In addition, new analyses of the predictive ability of these adjustments should be made in databases with less loss of SpO_2_ data and the new proposals should be validated in trauma systems at different levels of maturity, as well as in patients with penetrating trauma, since in this study this trauma mechanism was infrequent.

## Conclusions

This study proposed adjustments to the TRISS, which resulted in three new models of survival probability for trauma victims: NTRISS-like, TRISS SpO_2_, and NTRISS-like SpO_2_. The new models demonstrated accuracies above 89.0% and similarity of performance among themselves. Moreover, they displayed similar discriminatory capacity compared to that of the original and TRISS adjusted to the study population. These results suggest the potential for professionals to choose a model of survival probability that involves variables that are routinely measured and recorded, as SpO_2_. Most importantly, this PIPS tool meets service needs and is easy to use. Since all models have similar accuracy, one could choose the one that contains the variables that make the most sense to the local reality.
